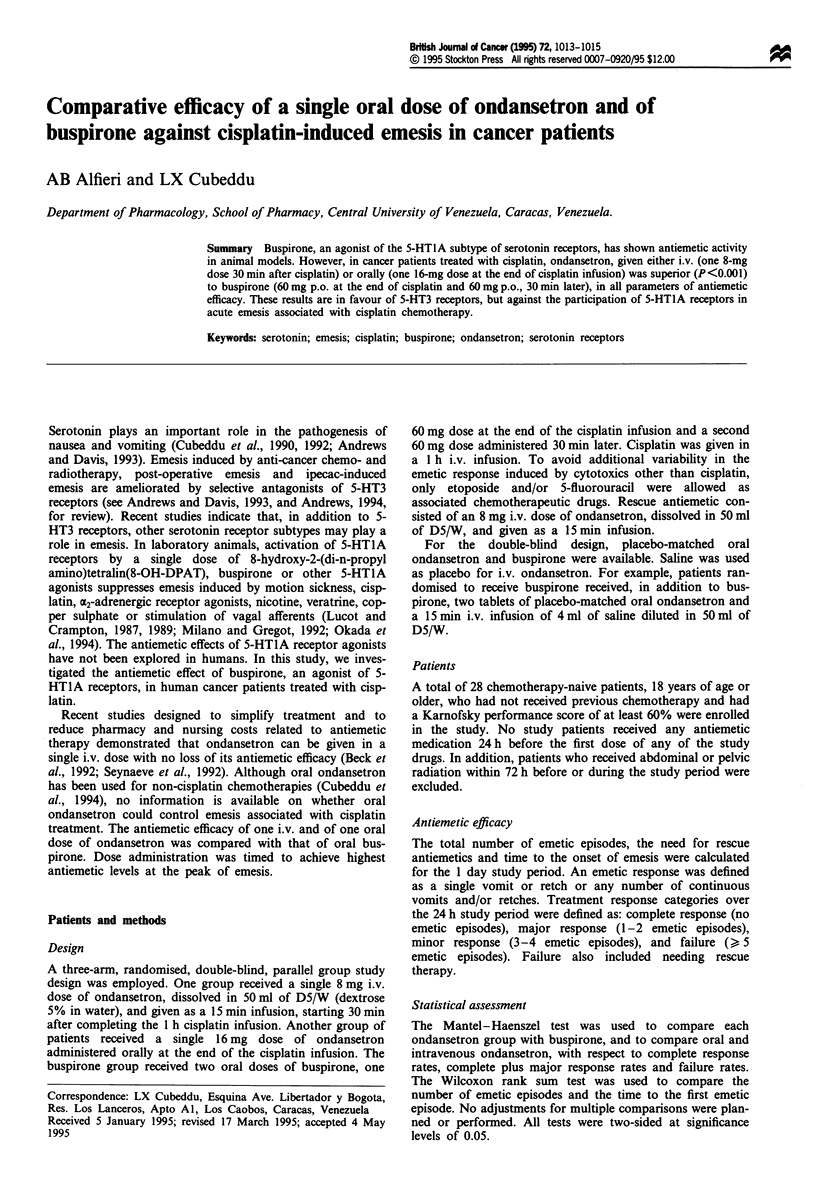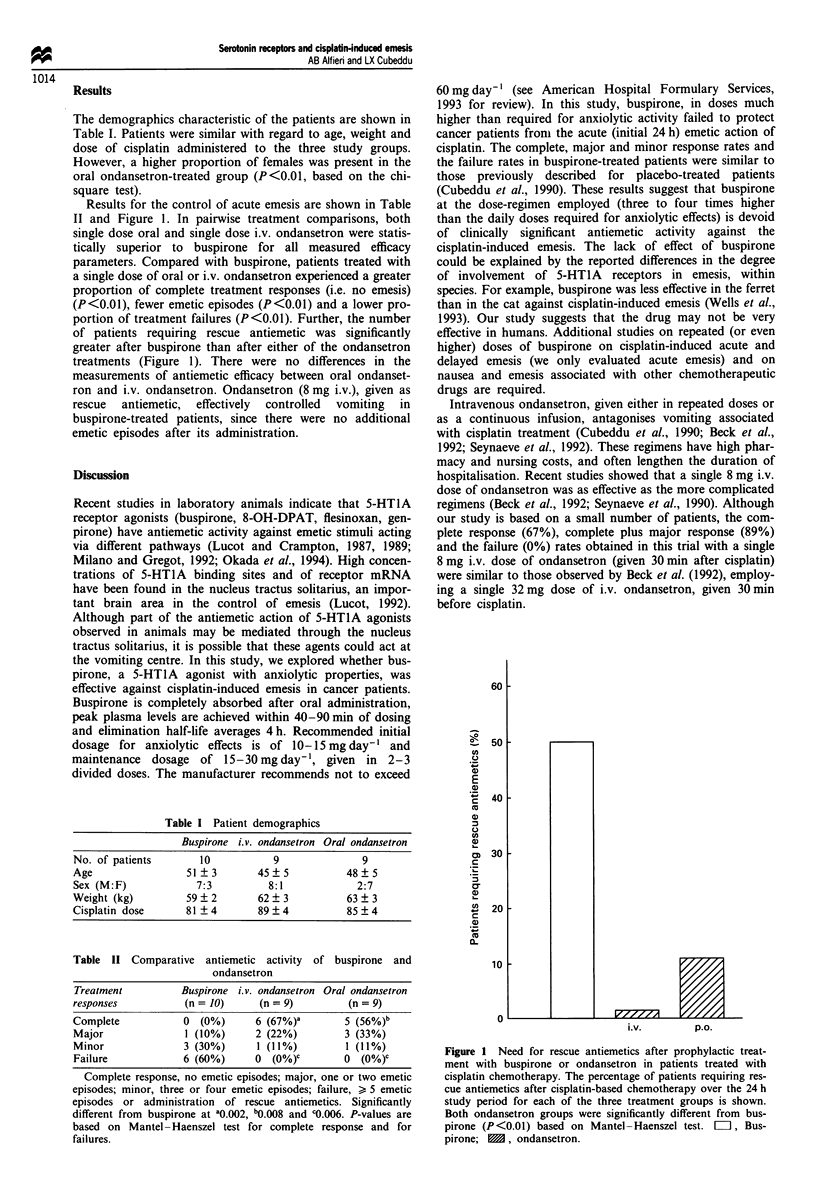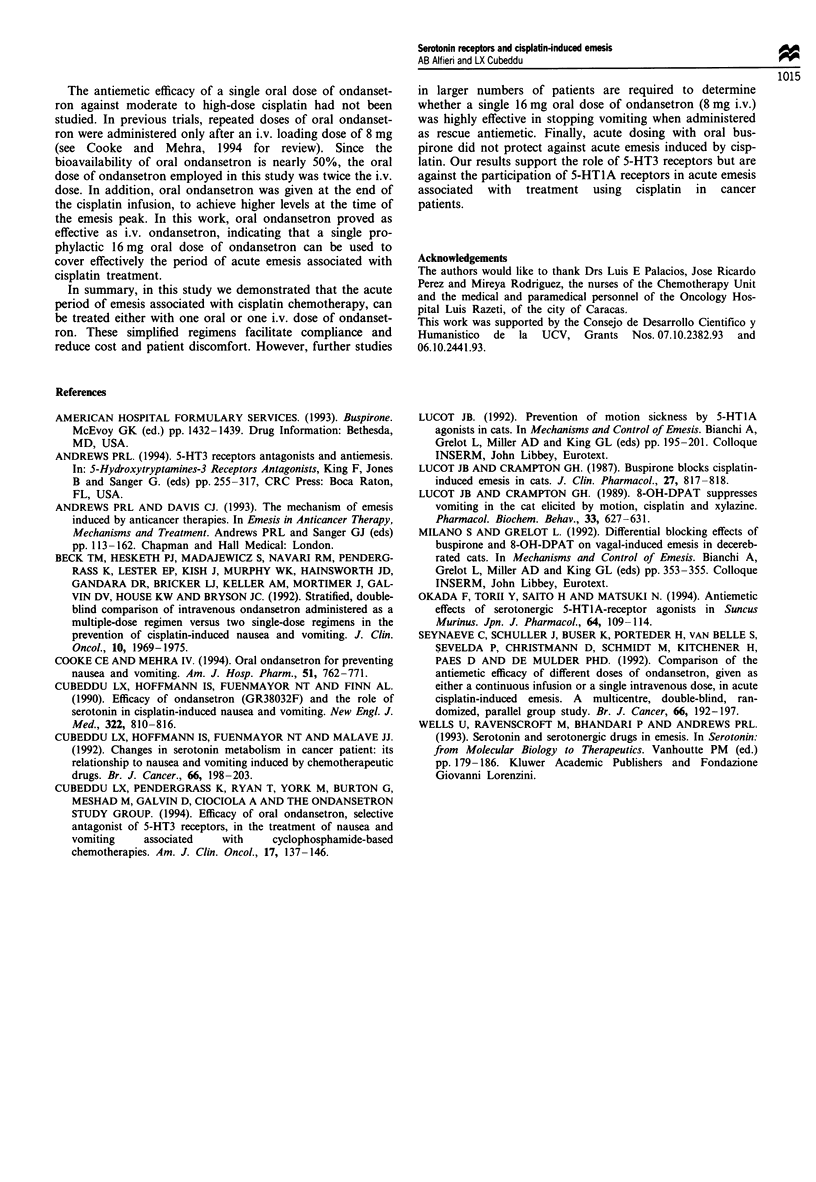# Comparative efficacy of a single oral dose of ondansetron and of buspirone against cisplatin-induced emesis in cancer patients.

**DOI:** 10.1038/bjc.1995.452

**Published:** 1995-10

**Authors:** A. B. Alfieri, L. X. Cubeddu

**Affiliations:** Department of Pharmacology, School of Pharmacy, Central University of Venezuela, Caracas.

## Abstract

Buspirone, an agonist of the 5-HT1A subtype of serotonin receptors, has shown antiemetic activity in animal models. However, in cancer patients treated with cisplatin, ondansetron, given either i.v. (one 8-mg dose 30 min after cisplatin) or orally (one 16-mg dose at the end of cisplatin infusion) was superior (P < 0.001) to buspirone (60 mg p.o. at the end of cisplatin and 60 mg p.o., 30 min later), in all parameters of antiemetic efficacy. These results are in favour of 5-HT3 receptors, but against the participation of 5-HT1A receptors in acute emesis associated with cisplatin chemotherapy.


British Journal of Cancer (1995) 72, 1013-1015
© 1995 Stockton Press All rights reserved 0007-0920/95 $12.00

### Comparative efficacy of a single oral dose of ondansetron and of buspirone against cisplatin-induced emesis in cancer patients

AB Alfieri and LX Cubeddu

*Department of Pharmacology, School of Pharmacy, Central University of Venezuela, Caracas, Venezuela.*

**Summary** Buspirone, an agonist of the 5-HT1A subtype of serotonin receptors, has shown antiemetic activity in animal models. However, in cancer patients treated with cisplatin, ondansetron, given either i.v. (one 8-mg dose 30 min after cisplatin) or orally (one 16-mg dose at the end of cisplatin infusion) was superior ($P<0.001$) to buspirone (60 mg p.o. at the end of cisplatin and 60 mg p.o., 30 min later), in all parameters of antiemetic efficacy. These results are in favour of 5-HT3 receptors, but against the participation of 5-HT1A receptors in acute emesis associated with cisplatin chemotherapy.

**Keywords:** serotonin; emesis; cisplatin; buspirone; ondansetron; serotonin receptors

Serotonin plays an important role in the pathogenesis of nausea and vomiting (Cubeddu *et al.*, 1990, 1992; Andrews and Davis, 1993). Emesis induced by anti-cancer chemo- and radiotherapy, post-operative emesis and ipecac-induced emesis are ameliorated by selective antagonists of 5-HT3 receptors (see Andrews and Davis, 1993, and Andrews, 1994, for review). Recent studies indicate that, in addition to 5-HT3 receptors, other serotonin receptor subtypes may play a role in emesis. In laboratory animals, activation of 5-HT1A receptors by a single dose of 8-hydroxy-2-(di-n-propyl amino)tetralin(8-OH-DPAT), buspirone or other 5-HT1A agonists suppresses emesis induced by motion sickness, cisplatin, $\alpha_2$-adrenergic receptor agonists, nicotine, veratrine, copper sulphate or stimulation of vagal afferents (Lucot and Crampton, 1987, 1989; Milano and Gregot, 1992; Okada *et al.*, 1994). The antiemetic effects of 5-HT1A receptor agonists have not been explored in humans. In this study, we investigated the antiemetic effect of buspirone, an agonist of 5-HT1A receptors, in human cancer patients treated with cisplatin.

Recent studies designed to simplify treatment and to reduce pharmacy and nursing costs related to antiemetic therapy demonstrated that ondansetron can be given in a single i.v. dose with no loss of its antiemetic efficacy (Beck *et al.*, 1992; Seynaeve *et al.*, 1992). Although oral ondansetron has been used for non-cisplatin chemotherapies (Cubeddu *et al.*, 1994), no information is available on whether oral ondansetron could control emesis associated with cisplatin treatment. The antiemetic efficacy of one i.v. and of one oral dose of ondansetron was compared with that of oral buspirone. Dose administration was timed to achieve highest antiemetic levels at the peak of emesis.

### Patients and methods

### *Design*

A three-arm, randomised, double-blind, parallel group study design was employed. One group received a single 8 mg i.v. dose of ondansetron, dissolved in 50 ml of D5/W (dextrose 5% in water), and given as a 15 min infusion, starting 30 min after completing the 1 h cisplatin infusion. Another group of patients received a single 16 mg dose of ondansetron administered orally at the end of the cisplatin infusion. The buspirone group received two oral doses of buspirone, one 60 mg dose at the end of the cisplatin infusion and a second 60 mg dose administered 30 min later. Cisplatin was given in a 1 h i.v. infusion. To avoid additional variability in the emetic response induced by cytotoxics other than cisplatin, only etoposide and/or 5-fluorouracil were allowed as associated chemotherapeutic drugs. Rescue antiemetic consisted of an 8 mg i.v. dose of ondansetron, dissolved in 50 ml of D5/W, and given as a 15 min infusion.

For the double-blind design, placebo-matched oral ondansetron and buspirone were available. Saline was used as placebo for i.v. ondansetron. For example, patients randomised to receive buspirone received, in addition to buspirone, two tablets of placebo-matched oral ondansetron and a 15 min i.v. infusion of 4 ml of saline diluted in 50 ml of D5/W.

### *Patients*

A total of 28 chemotherapy-naive patients, 18 years of age or older, who had not received previous chemotherapy and had a Karnofsky performance score of at least 60% were enrolled in the study. No study patients received any antiemetic medication 24 h before the first dose of any of the study drugs. In addition, patients who received abdominal or pelvic radiation within 72 h before or during the study period were excluded.

### *Antiemetic efficacy*

The total number of emetic episodes, the need for rescue antiemetics and time to the onset of emesis were calculated for the 1 day study period. An emetic response was defined as a single vomit or retch or any number of continuous vomits and/or retches. Treatment response categories over the 24 h study period were defined as: complete response (no emetic episodes), major response (1–2 emetic episodes), minor response (3–4 emetic episodes), and failure ($\geqslant 5$ emetic episodes). Failure also included needing rescue therapy.

### *Statistical assessment*

The Mantel–Haenszel test was used to compare each ondansetron group with buspirone, and to compare oral and intravenous ondansetron, with respect to complete response rates, complete plus major response rates and failure rates. The Wilcoxon rank sum test was used to compare the number of emetic episodes and the time to the first emetic episode. No adjustments for multiple comparisons were planned or performed. All tests were two-sided at significance levels of 0.05.

Correspondence: LX Cubeddu, Esquina Ave. Libertador y Bogota, Res. Los Lanceros, Apto A1, Los Caobos, Caracas, Venezuela
Received 5 January 1995; revised 17 March 1995; accepted 4 May 1995

### Results

The demographics characteristic of the patients are shown in Table I. Patients were similar with regard to age, weight and dose of cisplatin administered to the three study groups. However, a higher proportion of females was present in the oral ondansetron-treated group ($P<0.01$, based on the chi-square test).

Results for the control of acute emesis are shown in Table II and Figure 1. In pairwise treatment comparisons, both single dose oral and single dose i.v. ondansetron were statistically superior to buspirone for all measured efficacy parameters. Compared with buspirone, patients treated with a single dose of oral or i.v. ondansetron experienced a greater proportion of complete treatment responses (i.e. no emesis) ($P<0.01$), fewer emetic episodes ($P<0.01$) and a lower proportion of treatment failures ($P<0.01$). Further, the number of patients requiring rescue antiemetic was significantly greater after buspirone than after either of the ondansetron treatments (Figure 1). There were no differences in the measurements of antiemetic efficacy between oral ondansetron and i.v. ondansetron. Ondansetron (8 mg i.v.), given as rescue antiemetic, effectively controlled vomiting in buspirone-treated patients, since there were no additional emetic episodes after its administration.

### Discussion

Recent studies in laboratory animals indicate that 5-HT1A receptor agonists (buspirone, 8-OH-DPAT, flesinoxan, genpirone) have antiemetic activity against emetic stimuli acting via different pathways (Lucot and Crampton, 1987, 1989; Milano and Gregot, 1992; Okada *et al.*, 1994). High concentrations of 5-HT1A binding sites and of receptor mRNA have been found in the nucleus tractus solitarius, an important brain area in the control of emesis (Lucot, 1992). Although part of the antiemetic action of 5-HT1A agonists observed in animals may be mediated through the nucleus tractus solitarius, it is possible that these agents could act at the vomiting centre. In this study, we explored whether buspirone, a 5-HT1A agonist with anxiolytic properties, was effective against cisplatin-induced emesis in cancer patients. Buspirone is completely absorbed after oral administration, peak plasma levels are achieved within 40–90 min of dosing and elimination half-life averages 4 h. Recommended initial dosage for anxiolytic effects is of 10–15 mg $day^{-1}$ and maintenance dosage of 15–30 mg $day^{-1}$, given in 2–3 divided doses. The manufacturer recommends not to exceed 60 mg $day^{-1}$ (see American Hospital Formulary Services, 1993 for review). In this study, buspirone, in doses much higher than required for anxiolytic activity failed to protect cancer patients from the acute (initial 24 h) emetic action of cisplatin. The complete, major and minor response rates and the failure rates in buspirone-treated patients were similar to those previously described for placebo-treated patients (Cubeddu *et al.*, 1990). These results suggest that buspirone at the dose-regimen employed (three to four times higher than the daily doses required for anxiolytic effects) is devoid of clinically significant antiemetic activity against the cisplatin-induced emesis. The lack of effect of buspirone could be explained by the reported differences in the degree of involvement of 5-HT1A receptors in emesis, within species. For example, buspirone was less effective in the ferret than in the cat against cisplatin-induced emesis (Wells *et al.*, 1993). Our study suggests that the drug may not be very effective in humans. Additional studies on repeated (or even higher) doses of buspirone on cisplatin-induced acute and delayed emesis (we only evaluated acute emesis) and on nausea and emesis associated with other chemotherapeutic drugs are required.

Intravenous ondansetron, given either in repeated doses or as a continuous infusion, antagonises vomiting associated with cisplatin treatment (Cubeddu *et al.*, 1990; Beck *et al.*, 1992; Seynaeve *et al.*, 1992). These regimens have high pharmacy and nursing costs, and often lengthen the duration of hospitalisation. Recent studies showed that a single 8 mg i.v. dose of ondansetron was as effective as the more complicated regimens (Beck *et al.*, 1992; Seynaeve *et al.*, 1990). Although our study is based on a small number of patients, the complete response (67%), complete plus major response (89%) and the failure (0%) rates obtained in this trial with a single 8 mg i.v. dose of ondansetron (given 30 min after cisplatin) were similar to those observed by Beck *et al.* (1992), employing a single 32 mg dose of i.v. ondansetron, given 30 min before cisplatin.

**Table I** Patient demographics

| | *Buspirone* | *i.v. ondansetron* | *Oral ondansetron* |
|---|---|---|---|
| No. of patients | 10 | 9 | 9 |
| Age | 51 ± 3 | 45 ± 5 | 48 ± 5 |
| Sex (M:F) | 7:3 | 8:1 | 2:7 |
| Weight (kg) | 59 ± 2 | 62 ± 3 | 63 ± 3 |
| Cisplatin dose | 81 ± 4 | 89 ± 4 | 85 ± 4 |

**Table II** Comparative antiemetic activity of buspirone and ondansetron

| *Treatment responses* | *Buspirone* (n = *10*) | *i.v. ondansetron* (n = *9*) | *Oral ondansetron* (n = *9*) |
|---|---|---|---|
| Complete | 0 (0%) | 6 (67%)[a] | 5 (56%)[b] |
| Major | 1 (10%) | 2 (22%) | 3 (33%) |
| Minor | 3 (30%) | 1 (11%) | 1 (11%) |
| Failure | 6 (60%) | 0 (0%)[c] | 0 (0%)[c] |

Complete response, no emetic episodes; major, one or two emetic episodes; minor, three or four emetic episodes; failure, ⩾5 emetic episodes or administration of rescue antiemetics. Significantly different from buspirone at [a]0.002, [b]0.008 and [c]0.006. *P*-values are based on Mantel–Haenszel test for complete response and for failures.

**Figure 1** Need for rescue antiemetics after prophylactic treatment with buspirone or ondansetron in patients treated with cisplatin chemotherapy. The percentage of patients requiring rescue antiemetics after cisplatin-based chemotherapy over the 24 h study period for each of the three treatment groups is shown. Both ondansetron groups were significantly different from buspirone ($P<0.01$) based on Mantel–Haenszel test. ▭, Buspirone; ▨, ondansetron.

The antiemetic efficacy of a single oral dose of ondansetron against moderate to high-dose cisplatin had not been studied. In previous trials, repeated doses of oral ondansetron were administered only after an i.v. loading dose of 8 mg (see Cooke and Mehra, 1994 for review). Since the bioavailability of oral ondansetron is nearly 50%, the oral dose of ondansetron employed in this study was twice the i.v. dose. In addition, oral ondansetron was given at the end of the cisplatin infusion, to achieve higher levels at the time of the emesis peak. In this work, oral ondansetron proved as effective as i.v. ondansetron, indicating that a single prophylactic 16 mg oral dose of ondansetron can be used to cover effectively the period of acute emesis associated with cisplatin treatment.

In summary, in this study we demonstrated that the acute period of emesis associated with cisplatin chemotherapy, can be treated either with one oral or one i.v. dose of ondansetron. These simplified regimens facilitate compliance and reduce cost and patient discomfort. However, further studies in larger numbers of patients are required to determine whether a single 16 mg oral dose of ondansetron (8 mg i.v.) was highly effective in stopping vomiting when administered as rescue antiemetic. Finally, acute dosing with oral buspirone did not protect against acute emesis induced by cisplatin. Our results support the role of 5-HT3 receptors but are against the participation of 5-HT1A receptors in acute emesis associated with treatment using cisplatin in cancer patients.

**Acknowledgements**

The authors would like to thank Drs Luis E Palacios, Jose Ricardo Perez and Mireya Rodriguez, the nurses of the Chemotherapy Unit and the medical and paramedical personnel of the Oncology Hospital Luis Razeti, of the city of Caracas.

This work was supported by the Consejo de Desarrollo Cientifico y Humanistico de la UCV, Grants Nos. 07.10.2382.93 and 06.10.2441.93.

### References

AMERICAN HOSPITAL FORMULARY SERVICES. (1993). *Buspirone*. McEvoy GK (ed.) pp. 1432–1439. Drug Information: Bethesda, MD, USA.

ANDREWS PRL. (1994). 5-HT3 receptors antagonists and antiemesis. In: *5-Hydroxytryptamines-3 Receptors Antagonists*, King F, Jones B and Sanger G. (eds) pp. 255–317, CRC Press: Boca Raton, FL, USA.

ANDREWS PRL AND DAVIS CJ. (1993). The mechanism of emesis induced by anticancer therapies. In *Emesis in Anticancer Therapy, Mechanisms and Treatment*. Andrews PRL and Sanger GJ (eds) pp. 113–162. Chapman and Hall Medical: London.

BECK TM, HESKETH PJ, MADAJEWICZ S, NAVARI RM, PENDERGRASS K, LESTER EP, KISH J, MURPHY WK, HAINSWORTH JD, GANDARA DR, BRICKER LJ, KELLER AM, MORTIMER J, GALVIN DV, HOUSE KW AND BRYSON JC. (1992). Stratified, double-blind comparison of intravenous ondansetron administered as a multiple-dose regimen versus two single-dose regimens in the prevention of cisplatin-induced nausea and vomiting. *J. Clin. Oncol.*, **10**, 1969–1975.

COOKE CE AND MEHRA IV. (1994). Oral ondansetron for preventing nausea and vomiting. *Am. J. Hosp. Pharm.*, **51**, 762–771.

CUBEDDU LX, HOFFMANN IS, FUENMAYOR NT AND FINN AL. (1990). Efficacy of ondansetron (GR38032F) and the role of serotonin in cisplatin-induced nausea and vomiting. *New Engl. J. Med.*, **322**, 810–816.

CUBEDDU LX, HOFFMANN IS, FUENMAYOR NT AND MALAVE JJ. (1992). Changes in serotonin metabolism in cancer patient: its relationship to nausea and vomiting induced by chemotherapeutic drugs. *Br. J. Cancer.*, **66**, 198–203.

CUBEDDU LX, PENDERGRASS K, RYAN T, YORK M, BURTON G, MESHAD M, GALVIN D, CIOCIOLA A AND THE ONDANSETRON STUDY GROUP. (1994). Efficacy of oral ondansetron, selective antagonist of 5-HT3 receptors, in the treatment of nausea and vomiting associated with cyclophosphamide-based chemotherapies. *Am. J. Clin. Oncol.*, **17**, 137–146.

LUCOT JB. (1992). Prevention of motion sickness by 5-HT1A agonists in cats. In *Mechanisms and Control of Emesis*. Bianchi A, Grelot L, Miller AD and King GL (eds) pp. 195–201. Colloque INSERM, John Libbey, Eurotext.

LUCOT JB AND CRAMPTON GH. (1987). Buspirone blocks cisplatin-induced emesis in cats. *J. Clin. Pharmacol.*, **27**, 817–818.

LUCOT JB AND CRAMPTON GH. (1989). 8-OH-DPAT suppresses vomiting in the cat elicited by motion, cisplatin and xylazine. *Pharmacol. Biochem. Behav.*, **33**, 627–631.

MILANO S AND GRELOT L. (1992). Differential blocking effects of buspirone and 8-OH-DPAT on vagal-induced emesis in decerebrated cats. In *Mechanisms and Control of Emesis*. Bianchi A, Grelot L, Miller AD and King GL (eds) pp. 353–355. Colloque INSERM, John Libbey, Eurotext.

OKADA F, TORII Y, SAITO H AND MATSUKI N. (1994). Antiemetic effects of serotonergic 5-HT1A-receptor agonists in *Suncus Murinus*. *Jpn. J. Pharmacol.*, **64**, 109–114.

SEYNAEVE C, SCHULLER J, BUSER K, PORTEDER H, VAN BELLE S, ŞEVELDA P, CHRISTMANN D, SCHMIDT M, KITCHENER H, PAES D AND DE MULDER PHD. (1992). Comparison of the antiemetic efficacy of different doses of ondansetron, given as either a continuous infusion or a single intravenous dose, in acute cisplatin-induced emesis. A multicentre, double-blind, randomized, parallel group study. *Br. J. Cancer*, **66**, 192–197.

WELLS U, RAVENSCROFT M, BHANDARI P AND ANDREWS PRL. (1993). Serotonin and serotonergic drugs in emesis. In *Serotonin: from Molecular Biology to Therapeutics*. Vanhoutte PM (ed.) pp. 179–186. Kluwer Academic Publishers and Fondazione Giovanni Lorenzini.